# A New Al_2_O_3_‐Protected EB‐PVD TBC with Superior CMAS Resistance

**DOI:** 10.1002/advs.202305479

**Published:** 2023-12-03

**Authors:** Shiying Qin, Huatang Cao, Zhaohe Gao, Gyn Brewster, João P. Martin, Ying Chen, Ping Xiao

**Affiliations:** ^1^ Department of Materials and Henry Royce Institute University of Manchester Manchester M13 9PL UK; ^2^ Rolls‐Royce plc PO. Box 31 Derby DE24 8BJ UK

**Keywords:** CMAS, nanoporous Al_2_O_3_, sintering resistance, TBCs

## Abstract

Calcium‐magnesium‐aluminium‐silicate (CMAS) attack is a longstanding challenge for yttria stabilized zirconia (YSZ) thermal barrier coatings (TBCs) particularly at higher engine operating temperature. Here, a novel microstructural design is reported for YSZ TBCs to mitigate CMAS attack. The design is based on a drip coating method that creates a thin layer of nanoporous Al_2_O_3_ around YSZ columnar grains produced by electron beam physical vapor deposition (EB‐PVD). The nanoporous Al_2_O_3_ enables fast crystallization of CMAS melt close to the TBC surface, in the inter‐columnar gaps, and on the column walls, thereby suppressing CMAS infiltration and preventing further degradation of the TBCs due to CMAS attack. Indentation and three‐point beam bending tests indicate that the highly porous Al_2_O_3_ only slightly stiffens the TBC but offers superior resistance against sintering in long‐term thermal exposure by reducing the intercolumnar contact. This work offers a new pathway for designing novel TBC architecture with excellent CMAS resistance, strain tolerance, and sintering resistance, which also points out new insight for assembly nanoporous ceramic in traditional ceramic structure for integrated functions.

## Graphical abstract

This study introduces a novel microstructural design for yttria‐stabilized zirconia (YSZ) thermal barrier coatings (TBCs) to resist calcium‐magnesium‐aluminum‐silicate (CMAS) attack. It employs a drip coating method to create a thin nanoporous Al_2_O_3_ layer around YSZ columnar grains, enabling fast crystallization of molten CMAS close to the TBC surface. This approach suppresses CMAS infiltration and prevents further degradation of TBC structure.

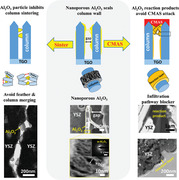

## 1 Introduction

Thermal barrier coatings (TBCs) made of Y_2_O_3_‐stabilized ZrO_2_ (YSZ) have been widely used in modern gas‐turbine engines to provide thermal protection for hot‐section metallic components^[^ ^1^, ^2^, ^3^, ^4^ ^]^ Over the past few decades, airborne contaminants have emerged as a significant factor contributing to early‐life failures in jet engines, especially when they operate in dusty environments such as volcanic plumes, sandstorms, and deserts.^[^ ^5^, ^6^, ^7^, ^8^ ^]^ The airborne contaminants are primarily composed of amorphous calcium‐magnesium‐alumino‐silicates (CMAS) and can melt inside the hot engines when heated above 1200 °C^[^ ^9^ ^]^). The molten CMAS infiltrates into the porous TBCs microstructure through capillary action and destabilizes the tough YSZ composition by thermochemical reactions. Upon cooling, CMAS solidifies within the TBCs microstructure, stiffens the coating by locking the strain‐tolerant microstructural features, and elevates the thermal stress in the coating, which consequently results in an increase in driving forces for TBCs failure and premature spallation.

Numerous approaches have been proposed to mitigate CMAS attack to YSZ TBCs,^[^ ^10^, ^11^, ^12^, ^13^, ^14^, ^15^ ^]^ mostly aiming to introduce dopants to immobilize CMAS or optimize coating structure.^[^ ^16^, ^17^, ^18^ ^]^ Al_2_O_3_ stands out for its promising CMAS resistance due to its ability to combine with major CMAS components (CaO and SiO_2_) to form anorthite and devitrify CMAS.^[^ ^19^, ^20^ ^]^ Another advantage of Al_2_O_3_ is its immiscibility with YSZ,^[^ ^21^ ^]^ which ensures the YSZ‐Al_2_O_3_ system is thermodynamically stable in the operating conditions of TBCs. But the challenge for integrating Al_2_O_3_ into TBCs is to engineer durable TBCs architecture. Traditional routes include implementing Al_2_O_3_ as a metastable solid solution^[^ ^22^, ^23^ ^]^ or an exterior protection layer in TBCs systems.^[^ ^12^, ^24^ ^]^ However, the Al_2_O_3_ alloyed YSZ is thermodynamically unstable and undergoes phase destabilization (*t*‐ZrO_2_ to *m*‐ZrO_2_) because of metastable Al solute diffusion out from *t*‐ZrO_2_ during thermal treatment.^[^ ^25^, ^26^ ^]^ In addition, with the application of the Al_2_O_3_ overlay on the top of TBCs, spallation tends to be generated at the Al_2_O_3_ overlay/TBCs interface due to thermal expansion misfit between Al_2_O_3_ and YSZ.^[^ ^27^ ^]^ Those drawbacks thereby pose a great impact on the efficiency against CMAS attack.

In this work, we designed a novel Al_2_O_3_‐protected EB‐PVD TBC architecture to withstand CMAS attack, where individual EB‐PVD columns are coated with nanoporous Al_2_O_3_. The design is inspired by the well‐established CMAS attack mechanism that CMAS penetration into EB‐PVD TBCs is through their inter‐columnar gaps. Such a mechanism suggests an efficient CMAS mitigation strategy is to decorate these inter‐columnar gaps with a thin layer of CMAS‐resistant material (e.g., Al_2_O_3_). Meanwhile, as the design exclusively targets the vulnerable CMAS infiltration pathways, the high robustness of the YSZ TBCs is expected to be retained as much as possible. Al_2_O_3_ was selected in this work due to its ability to immobilize CMAS and its thermodynamic compatibility with YSZ. The nanoporous Al_2_O_3_‐protected EB‐PVD TBCs are fabricated by a simple and low‐cost vacuum‐assisted drip coating method (VADC).^[^ ^28^ ^]^ This method is a post‐process treatment technique, which simplifies the manufacturing process and can be potentially used for commercial production. Investigations were focused on the microstructure and phase variation of TBCs induced by CMAS attack. Meanwhile, the effect of Al_2_O_3_ on the stiffness and sintering of TBCs was examined by both mechanical and microstructure characterizations.

## 2 Experimental Section

### 2.1 Drip Coating Process

The materials used in this work were in the form of a turbine blade which comprises a CMSX‐4 single crystal superalloy substrate, a platinum aluminide bond coat, and an 8 wt.% Y_2_O_3_‐stabilized ZrO_2_ (YSZ) topcoat deposited by electron beam physical vapor deposition (EB‐PVD). The thickness of the YSZ topcoat is ≈120 µm. Rectangular samples (1 cm ×1 cm × 0.2 cm) were cut from the trailing edge of the turbine blade by a precision cut‐off machine. The samples were cleaned with a mixture solution (acetone: ethanol = 1:1 in volume) by ultrasonic vibration for 10 min and dried in an oven at 60 °C.

Before drip coating, AlCl_3_ sol was prepared by using AlCl_3_. 6H_2_O (Aldrich, 99%) as the precursor material. The AlCl_3_. 6H_2_O was dissolved in a mixture of deionized water and ethanol (water: ethanol = 0.72:1 in volume), followed by stirring for 30 min in order to obtain a homogeneous solution. After that, the solution was applied onto the EB‐PVD TBCs using the drip coating method.

**Figure 1:**
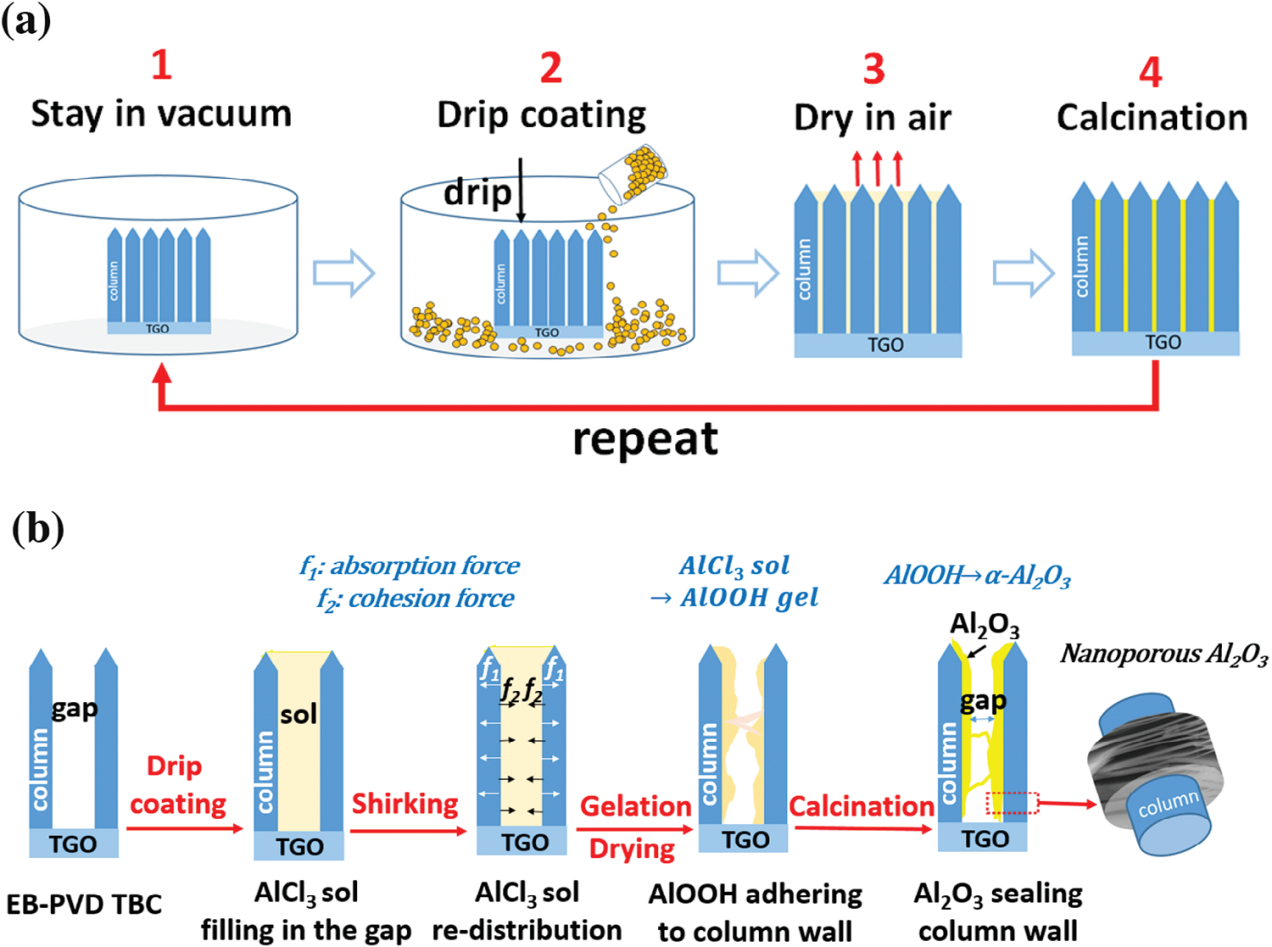
Illustration diagram for a) the drip coating process; b) the formation process of nanoporous Al_2_O_3_ in TBCs.

A schematic of the drip coating process is shown in **Figure** **1**. The EB‐PVD TBCs and AlCl_3_ sol (contained in a beaker) were put into a castable vacuum system (Cast N'Vac 1000). The system was then pre‐vacuumed to ≈10^4^ Pa for 5 min. Once the pressure was stabilized, the AlCl_3_ sol was slowly dripped on the coating surface until it entirely submerged the coating. The samples were kept in vacuum for another 15 min to ensure sufficient infiltration of AlCl_3_ sol inside the coatings. The drip‐coated samples were then kept at 40 °C to allow the gelation of the infiltrated sol. After gelation, the samples with wet gel inside were aged for 24 h and evaporatively dried at 40 °C in an ambient air environment.^[^ ^29^ ^]^ Then, the drip‐coated samples were placed into a tube furnace (Lenton Tube Furnace 1200 °C) and calcinated at 1000 °C for 1 h to convert the implanted gel into alumina. The course was regarded as drip coating for one time. This process could be repeated to control the alumina distribution (e.g., the infiltration depth and amount of alumina) inside coatings, which provides an opportunity to develop an optimal coating microstructure. In this work, EB‐PVD TBCs drip‐coated was fabricated for different times (1–6). All the characterizations for Al_2_O_3_‐protected TBCs were carried out after the calcination step.

### 2.2 CMAS Attack

To evaluate the efficiency of Al_2_O_3_‐protected EB‐PVD TBCs against CMAS attack, coatings with and without Al_2_O_3_ were exposed to CMAS attack under the same condition. The chemical composition of the CMAS used in the experiment was 35CaO–10MgO–7Al_2_O_3_–48SiO_2_ (in mole percent), and the liquidus temperature of the CMAS is 1235 °C.^[^ ^30^ ^]^ The CMAS testing temperature was selected as 1250 °C. This temperature was above the melting point of the CMAS but was not expected to cause excessive intrinsic degradation of YSZ‐based TBCs,^[^ ^31^ ^]^ thereby providing an opportunity for studying CMAS‐induced damage to the TBCs. Different durations at the testing temperature were used (*t* = 10 min, 30 min, and 2 h) to examine the effectiveness of Al_2_O_3_ on CMAS attack, both in the short‐term and long‐term. The CMAS powder was pre‐mixed with ethanol and loaded into a syringe. The mixture was then slowly dripped on the coating surface by the syringe. The dose of CMAS powder was controlled to be 40 mg cm^−2^. After that, the CMAS‐covered coatings were dried in an oven at 80 °C and then transferred into a muffle furnace (Carbolite gero RHF 1400) and treated at 1250 °C for different durations as mentioned above. Both the heating and cooling rates applied in this study were 10 °C min^−1^. The heat treatment applied in this study was isothermal. It had to be mentioned that the isothermal heat treatment could cause more severe CMAS attack to EB‐PVD TBCs, compared to what happens in a real situation where a thermal gradient exists through the coating.

### 2.3 Mechanical Properties

The Al_2_O_3_ that infiltrated into the EB‐PVD TBCs partially occupies the inter‐columnar gaps and will be involved in sintering in thermal exposure, which might affect the strain tolerance (stiffness) of the coatings. As a result, micro indentation, nano indentation, and three‐point beam bending tests were carried out to evaluate the effect of the built‐in Al_2_O_3_ on the Young's modulus of the coating across multiple length scales spanning from individual columns to the entire coating.

Depth‐instrumented indentation was performed by a micro‐indentation tester (MHT, Anton Paar) at room temperature. An indenter with Vickers geometry calibrated with a standard silica specimen was used by running a standard continuous stiffness measurement (CSM). The maximum penetration depth was set as 5 µm. Measurements were performed on the polished cross‐sections of EB‐PVD TBCs in different regions (referred as top, middle, and bottom regions), and for each region, 25 measurements were made. Nano‐indentation test was carried out on the polished cross‐sections of EB‐PVD TBCs to determine the in‐plane elastic modulus of individual columns. Measurements were performed in the top region of the cross‐section. A TI 950 nano‐indenter was used. For each sample, a 4 × 40 (5 µm step size) array of indents were made using a Berkovich indenter with a penetration depth of 200 nm. The elastic modulus was calculated from the slope of the unloading section of the load‐displacement curve by Oliver & Parr method.^[^ ^32^ ^]^

Three‐point beam bending tests were conducted on rectangular free‐standing TBCs with a dimension of ≈10 × 2.0 × 0.15 mm^3^ using a bend fixture with a span of 5 mm on an Instron universal testing machine. The maximum load of the test machine is 10 N. The free‐standing TBC beams were removed from the metal substrates by acid soaking (HCl: HNO_3_ = 3:1 in volume) for 24 h, and was confirmed to be ceramic bilayer (TBC+TGO). During the beam bending test, the YSZ side was faced upward (in compression) and the central loading point was placed on the YSZ side. At least five beams were tested for each condition. The Young's modulus of the bilayer beam (*E*_bi_)was extracted from the load‐displacement curves. For a bilayer composite material with a known Young's modulus *E*_bi_, the modulus of TBC (*E*_TBC_) can be calculated by following equations^[^ ^33^ ^]^:

(1) $$\begin{array}{c} E_{\mathrm{TBC}}\;=\;\frac{\frac{E_{\mathrm{bi}}{\left(h_{\mathrm{TGO}}+h_{\mathrm{TBC}}\right)}^{3}}{4}\;-\;E_{\mathrm{TGO}}\;\left[\;{\left(h_{\mathrm{TGO}}+h_{\mathrm{TBC}}-h_{0}\right)}^{3}-{\left(h_{\mathrm{TBC}}-h_{0}\right)}^{3}\right]}{h_{0}^{3}+{\left(h_{\mathrm{TBC}}-h_{0}\right)}^{3}} \end{array}$$

Where *E*_TGO_ is the Young's modulus of the TGO and is assumed to be 350 GPa.^[^ ^34^ ^]^ *h*_TGO_ and *h*_TBC_ are the thickness of the TGO and TBC layers respectively, which were measured by SEM. *h*_0_ is the height of the neutral axis when the bilayer is loaded:

(2) $$h_{0}=\frac{E_{\mathrm{TGO}}h_{\mathrm{TGO}}^{2}+E_{\mathrm{TBC}}h_{\mathrm{TBC}}^{2}+2E_{\mathrm{TGO}}h_{\mathrm{TGO}}h_{\mathrm{TBC}}}{2E_{\mathrm{TGO}}h_{\mathrm{TGO}}+2E_{\mathrm{TBC}}h_{\mathrm{TBC}}}$$

To investigate the sintering behavior of Al_2_O_3_‐protected EB‐PVD TBCs, samples with and without Al_2_O_3_ were both isothermally treated at 1150 °C for different durations (*t* = 2, 50, and 100 h). 1150 °C is the temperature usually used for YSZ sintering based on previous reports.^[^ ^35^, ^36^ ^]^ Since the densification of EB‐PVD TBCs structure can be indirectly reflected by its Young's modulus, both micro and nano indentations were performed on the polished cross section of TBCs before and after sintering. The characterization areas were in the top regions of the TBC cross sections, and depth‐instrumented indentation was employed in the same way as mentioned above. In the meantime, three‐point beam bending tests were conducted to reveal the sintering behavior of Al_2_O_3_‐protected EB‐PVD TBCs at the macroscopic scale.

### 2.4 Characterizations

The phase structures of the coatings near the surface regions before and after CMAS attack were studied by X‐ray diffraction (XRD, X'Pert Pro, Malvern Panalytical) with Cu Kα radiation (Kα1 = 1.540598 Å). The diffraction data were collected by a continuous scan mode in a 2θ range of 10^o^–90^o^. Photoluminescence piezo‐spectroscopy (PLPS) based on a Raman microscope (Renishaw inVia, He‐Ni 633 nm) was used to identify the phase structures of Al_2_O_3_ infiltrated in EB‐PVD TBCs according to the methodology described in the literature.^[^ ^37^ ^]^ PLPS measurements were taken from the polished cross‐sections of the coatings prepared by standard metallographic procedures. The laser was focused on column gaps since Al_2_O_3_ was supposed to be mainly distributed in those areas. The spot size of the focused laser was in the range of ≈3–5 µm. The cross‐sectional microstructures of the TBCs were characterized by scanning electron microscopy (SEM, FEI, Quanta 650, Magellan HR, and Zeiss Merlin) equipped with an energy‐dispersive X‐ray spectroscopy (EDS) system. To study the microstructures and the compositions of the coatings and their reaction products with CMAS in greater detail, thin lamellae of Al_2_O_3_‐protected EB‐PVD TBCs before and after CMAS attack were prepared by a focused ion beam (FIB, FEI, Helios 660) and then examined by transmission electron microscopy (TEM, FEI, Tecnai T30; and Talos, F200A) fitted with EDS.

## 3 Results

### 3.1 Microstructure of Al_2_O_3_‐Protected EB‐PVD TBCs

#### 3.1.1 Al_2_O_3_ Phase Structure

**Figure 2:**
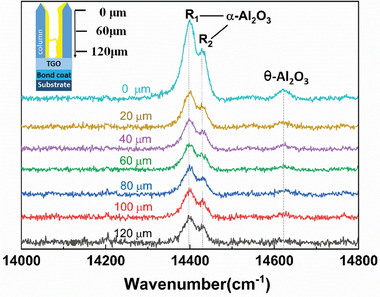
PLPS spectra of the internal Al_2_O_3_ of EB‐PVD TBCs after drip coating for one time, measured at different locations with a distance of 20 µm, starting from the top surface toward to coating/thermal growth oxide (TGO) interface as shown in the insert schematic of TBCs.

**Figure** **2** shows PLPS spectra of Al_2_O_3_‐protected TBCs after drip coating for one time. Measurements were performed at different locations along the coating thickness direction from the top surface to the coating/TGO interface as shown in the inserted schematic. The characteristic peaks of stress‐free α‐Al_2_O_3_ and θ‐Al_2_O_3_ are at 14402 and 14432, and 14575 and 14645 cm^−1^, respectively.^[^ ^37^, ^38^ ^]^ Based on these reference peak positions, characteristic R lines (R_1_ and R_2_) of α‐Al_2_O_3_ have been observed in PLPS spectra collected from the inter‐columnar gap, and minor peaks of metastable θ‐Al_2_O_3_ were also identified in the near surface region of TBCs. This finding suggests that α‐Al_2_O_3_ has been infiltrated in TBCs via drip coating. In addition, a higher peak intensity has been observed in the near surface region, thereby indicating a higher Al_2_O_3_ content in this region_._

#### 3.1.2 Al_2_O_3_ Distribution

**Figure 3:**
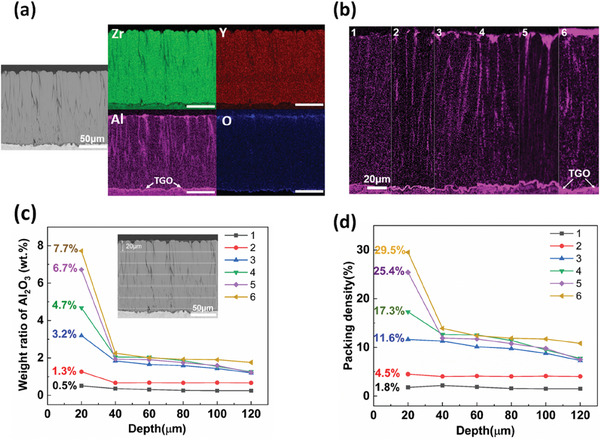
Compositional analysis of EB‐PVD YSZ TBCs after drip coating: a) cross sectional microstructure and EDS map of EB‐PVD YSZ TBCs drip coated for two times. The scale bars are 50 µm; b) Al map of EB‐PVD YSZ TBCs drip coated for different times (*t* = 1–6); c) Al_2_O_3_ weight ratio along coating cross section for samples drip coated for different times (*t* = 1–6). The Al_2_O_3_ weight ratio is calculated from the EDS data obtained along the coating cross section. The EDS scans were performed in 6 boxes, each spanning the entire length of image and having a width of 20 µm; d) packing density of Al_2_O_3_ along coating cross section for sample drip coated for different times (*t* = 1–6). The packing density of Al_2_O_3_ represents the volume ratio of injected Al_2_O_3_ to column gap, and calculation is based on the results of weight ratio of Al_2_O_3_ presented in image (c). The volume of column gap is equal to porosity and is assumed to be 30% and 20% in upper 20 µm region and 20–120 µm region, respectively.

**Figure** **3** shows the cross‐sectional microstructure and EDS analysis of Al_2_O_3_‐protected EB‐PVD TBCs. It can be seen clearly that Al exists throughout the entire coating, but its distribution is uneven along the coating depth (Figure 3a). A larger amount of Al_2_O_3_ is identified in the grooves between the columns near the coating surface, which is consistent with the stronger PLPS signals seen in Figure 2.

**Figure 4:**
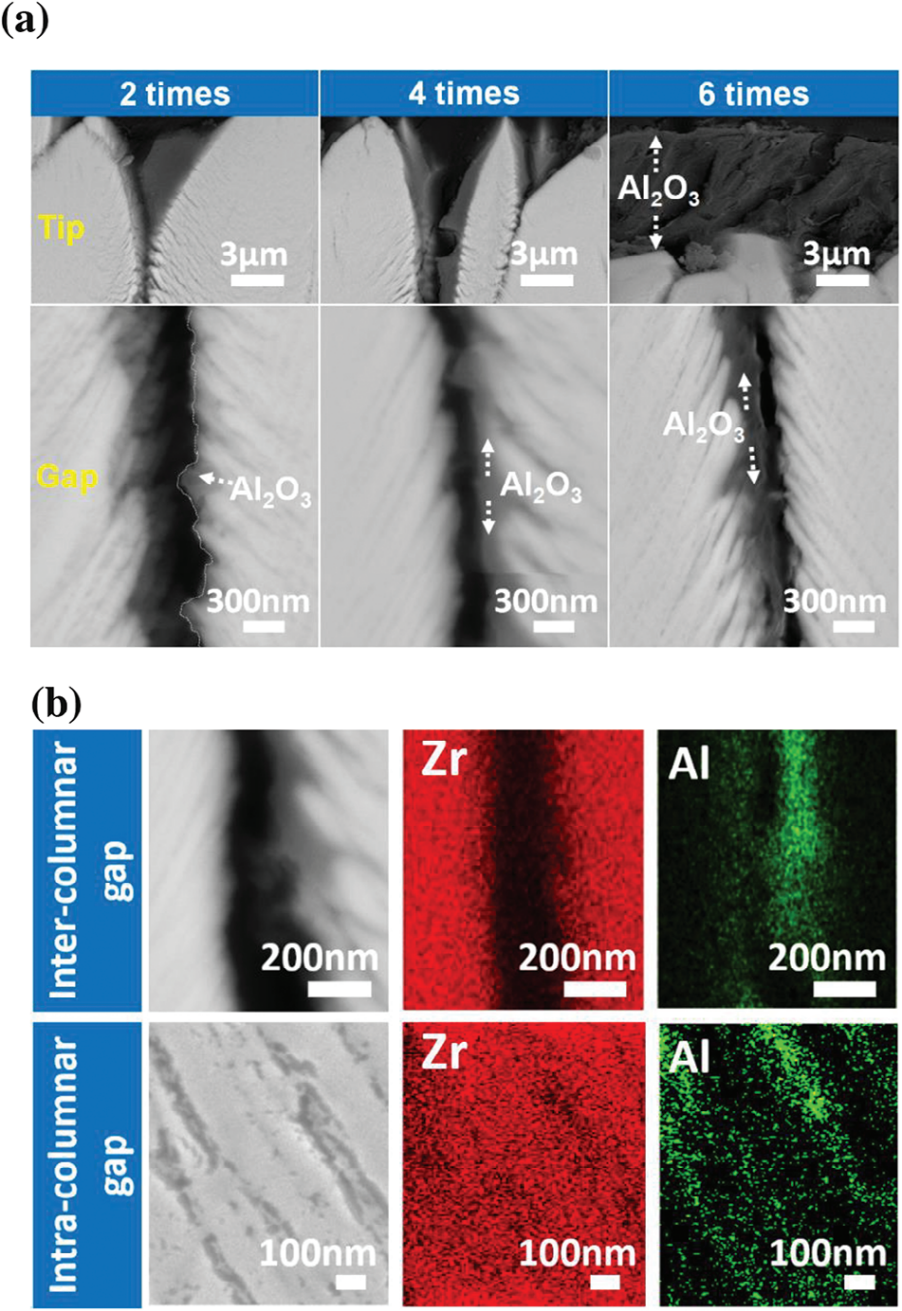
Microstructural analysis of EB‐PVD YSZ TBCs after drip coating: a) microstructure of EB‐PVD YSZ TBCs drip coated for different times (*t* = 2, 4, and 6), characterized by the circular backscattered electron (CBS) detector of a Magellan SEM at an accelerating voltage of 5 kV (Magellan, FEI). The areas shown are located in column tip and gap regions; b) microstructures and EDS maps of the inter‐columnar and intra‐columnar gaps of an EB‐PVD YSZ TBC drip coated for two times. The areas shown are located near the top coat/TGO interface region.

The cross‐sectional BSE micrographs in **Figure** **4**a compare the distribution of Al_2_O_3_ after a series of different drip coating times in both column tip and gap regions. It can be found that instead of filling up the column gaps, the injected Al_2_O_3_ is mainly distributed on column walls and the intra‐columnar gap (Figure 4b). The column grooves at the coating surface are gradually filled with Al_2_O_3_ until a thin layer has been formed above.

To estimate how much inter‐columnar space has been occupied by Al_2_O_3_ after drip coating, we determined the average Al_2_O_3_ content in selected areas through the coating thickness by quantitative EDS analysis and then converted the average Al_2_O_3_ content to a packing density. This analysis provides a measure for understanding the strain tolerance of Al_2_O_3_‐protected TBCs as it is determined by how much inter‐columnar space has been occupied by Al_2_O_3_ after drip coating. The evolution of Al_2_O_3_ content (weight ratio) with drip coating time is shown in Figure 3c. It can be seen clearly that the amount of Al_2_O_3_ injected into the coating in each drip is not equal. With the increase of drip coating time, a continuous increase of Al_2_O_3_ content has been observed in the near surface region (0–20 µm). However, as one moves away from the surface region to the inner part of the coating the content of Al_2_O_3_ levels off (≈2 wt.%) when drip coating time exceeds 3. This is probably caused by the build‐up of Al_2_O_3_ in column grooves which blocks the injection pathways in the subsequent cycle. The packing density of Al_2_O_3_ (*C_p_*) is defined as the volume ratio of Al_2_O_3_ to the column gap, which can be written as:

(3) $$C_{p}=\frac{V_{{\mathrm{Al}}_{2}O_{3}}}{V_{\mathrm{Gap}}}=\frac{V_{{\mathrm{Al}}_{2}O_{3}}/V_{\mathrm{coating}}}{V_{\mathrm{Gap}}/V_{\mathrm{coating}}}=\frac{{V^{\prime }}_{{\mathrm{Al}}_{2}O_{3}}}{{V^{\prime }}_{\mathrm{Gap}}}$$

where *V*_Gap_, $V_{{\mathrm{Al}}_{2}O_{3}}$, and *V*_coating_ are the volumes of column gap, Al_2_O_3_ in the column gap, and entire coating respectively; while *V*′_Gap_ and ${V^{\prime }}_{{\mathrm{Al}}_{2}O_{3}}$ are the volume ratios of column gap and Al_2_O_3_ in the column gap respectively.

The overall porosity of EB‐PVD TBCs is ≈20−30%,^[^ ^18^, ^39^, ^40^ ^]^ without regard to the intra‐columnar fine pores and voids between feather arms. Therefore, the volume ratio of column gap (*V*′_Gap_) in EB‐PVD TBCs is taken as a constant ranging from 20%−30%. The volume ratio of YSZ column (*V*′_YSZ_) equals 1 − *V*′_Gap_. Hence, the weight ratio of Al_2_O_3_ (Al_2_O_3_ wt. %) in EB‐PVD coating can be written as:

(4) $$\begin{array}{ccc} & & {\mathrm{Al}}_{2}O_{3}\mathrm{wt}.\%\\ & & \;\;=\frac{\rho_{{\mathrm{Al}}_{2}O_{3}}{V^{\prime }}_{{\mathrm{Al}}_{2}O_{3}}}{\rho_{{\mathrm{Al}}_{2}O_{3}}{V^{\prime }}_{{\mathrm{Al}}_{2}O_{3}}+\rho_{\mathrm{YSZ}}\;\left(1-{V^{\prime }}_{\mathrm{Gap}}\right)+\rho_{\mathrm{Gap}}\;\left({V^{\prime }}_{\mathrm{Gap}}-{V^{\prime }}_{{\mathrm{Al}}_{2}O_{3}}\right)} \end{array}$$

In which, ρ_YSZ_, ρ_Gap_ and $\rho_{{\mathrm{Al}}_{2}O_{3}}$ are the theoretical densities of 8YSZ, column gap and Al_2_O_3_, are taken as 5.97 g cm^−3^,^[^ ^41^ ^]^ 0 and 3.95 g cm^−3^, respectively. Insert Equations (4) into (3), we have:

(5) $$C_{p}=\frac{\rho_{\mathrm{YSZ}}\left(1-{V^{\prime }}_{\mathrm{Gap}}\right)}{\rho_{{\mathrm{Al}}_{2}O_{3}}{V^{\prime }}_{\mathrm{Gap}}}\cdot \left(\frac{{\mathrm{Al}}_{2}O_{3}\mathrm{wt}.\%}{1-{\mathrm{Al}}_{2}O_{3}\mathrm{wt}.\%}\right)$$

Here, *V*_Gap_ is assumed to be 30% in the upper 20 µm region and 20% in below 20–120 µm region, due to the relatively larger gap width in the near surface region of TBCs. Therefore, $\frac{\rho_{\mathrm{YSZ}}\left(1-{V^{\prime }}_{\mathrm{Gap}}\right)}{\rho_{{\mathrm{Al}}_{2}O_{3}}{V^{\prime }}_{\mathrm{Gap}}}$ is a constant at a given depth and the packing density of Al_2_O_3_ can be calculated by Al_2_O_3_ weight ratio shown in Figure 3c. It has to be mentioned that the calculated packing density is likely to be slightly underrated, since the YSZ columns are not fully dense (fine pores exist in YSZ columns).

Figure 3d shows the evolution of Al_2_O_3_ packing density along coating cross section. The variation trend of packing density is similar to that of the Al_2_O_3_ content shown in Figure 3c. Only a small volume fraction of the gaps (<5%) has been occupied by Al_2_O_3_ after drip coating for two times. However, a sudden increase in packing density occurs after drip coating for three times. A further increase in cycle number of drip coating to six times mostly increases the Al_2_O_3_ packing density near the surface region. The maximum packing densities in the upper region (0–20 µm) and coating internal (20–120 µm) are below 30% and 14% respectively. The results of the packing density indicate the injected Al_2_O_3_ only partially occupies the inter‐columnar gap. Therefore, it is expected that the high strain tolerance of EB‐PVD TBCs is mostly retained after the drip coating process. Further experiment evidence to support this argument will be presented in Section 3.2.1.

#### 3.1.3 Al_2_O_3_ Morphology

**Figure 5:**
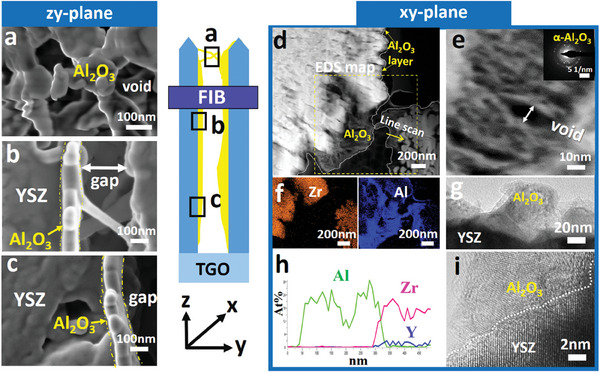
Microstructure and elemental distribution of EB‐PVD column after drip coated two times. SEM images of the nanoporous Al_2_O_3_ measured along the coating depth direction (*zy*‐plane of TBCs) at different locations: a) groove between the columns; b) halfway through the depth of a column; c) near the bottom of the column. Microstructures of Al_2_O_3_‐protected EB‐PVD column measured at the vertical direction of coating depth (*xy*‐plane of TBCs). The FIB slice is cut parallel to coating surface from the region enclosed by a blue rectangle. d) HAADF image of Al_2_O_3_‐protected EB‐PVD column. e) high‐resolution HAADF image of the nanoporous Al_2_O_3_, with an inset of its diffraction pattern; f) EDS elemental maps of the yellow dotted box in image (d); g) bright‐field TEM image of the thin Al_2_O_3_ layer on EB‐PVD column; h) EDS line scan acquired along the yellow arrow in image (d). The length of the scan was 480 nm; i) HRTEM image of YSZ/Al_2_O_3_ interface. The dark and bright regions represent YSZ and Al_2_O_3_, respectively.

Microstructural characterizations of Al_2_O_3_ were conducted along the vertical and horizontal directions of TBCs after drip coating for two times. **Figure** **5**a–c present the morphology of Al_2_O_3_ along the coating depth (*z*–*y* plane of TBCs), in which the built‐in Al_2_O_3_ is nanoporous (Figure 5a) and forms a nanoscale thin layer on the column wall (Figure 5b,c). To view the built‐in Al_2_O_3_ in finer detail, a thin lamella along the horizontal direction of the coating (x‐y plane of TBCs) was prepared by FIB and then analyzed by STEM, TEM and EDS. The characterization region is ≈60 µm below the coating surface, marked by the blue rectangle in the schematic in Figure 5. It can be found that a continuous Al_2_O_3_ layer is coated on the column wall, but most of the inter‐columnar porosity is retained (Figure 5d). The thickness of the Al_2_O_3_ layer varies from several hundred nanometers (≈100–300 nm, Figure 5d) to dozens of nanometers (≈20–50 nm, Figure 5g). The observation of the Al_2_O_3_ layer in both the *x*–*y* and *z*–*y* planes of TBCs indicates the high coverage of Al_2_O_3_ on the column wall. Additionally, nano voids with a size of ≈10 nm exist in the Al_2_O_3_ layer based on the HAADF image in Figure 5e.

The phase structure of the nanoporous Al_2_O_3_ is identified as α‐Al_2_O_3_ according to the diffraction patterns in Figure 5e. In Figure 5i, it can be found that the nanoporous Al_2_O_3_ is coated on the YSZ column without interfacial cracks. An EDS line scan was acquired from the Al_2_O_3_ to the column internals (Figure 5h). An overlap of elements Al and Zr has been observed at the interface region (with a thickness of 50 nm) between the nanoporous Al_2_O_3_ and the YSZ column, which also suggests no interfacial cracks exist between Al_2_O_3_ and column wall. The observed overlap is primarily attributed to the geometry effect of the YSZ column, which leads to an irregular shape of the YSZ in the *z*‐direction on the FIB slice. These results indicate the successful creation of nanoporous Al_2_O_3‐_protected TBCs architecture via the drip coating method.

### 3.2 Mechanical Properties

#### 3.2.1 Young's Modulus of Al_2_O_3_‐Protected EB‐PVD TBCs

**Figure 6:**
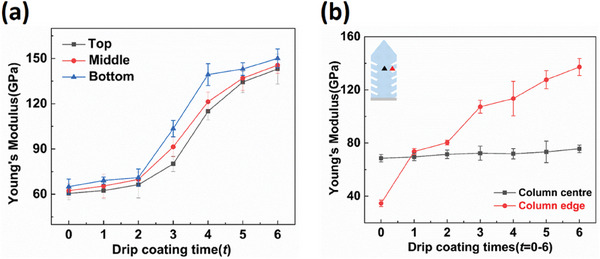
Young's modulus of Al_2_O_3_‐protected EB‐PVD TBCs as a function of drip coating times (*t* = 0–6). (a): Young's modulus of several columns conducted by micro indentation. Characterization has been performed at different regions (top, middle and bottom) along TBCs cross section; b) Young's modulus of column center and edge conducted by nano indentation. Characterization has been performed at the top region of TBCs cross section.

The injection of Al_2_O_3_ in EB‐PVD TBCs results in a reduction of coating porosity and the introduction of stiffer Al_2_O_3_. As a result, the Young's modulus of Al_2_O_3_‐protected TBCs is expected to increase. **Figure** **6**a compares the Young's modulus between the top, middle, and bottom regions of TBCs after drip coating for different times (0–6). The Young's modulus was determined by micro indentation performed on the cross section of TBCs at different coating depth. The evolution of Young's modulus with drip coating time is similar at different TBCs regions (top, middle, and bottom regions), while the bottom region always shows the highest value of Young's modulus in comparison to the other two regions for a certain drip coating time. This is because the bottom region has the lowest porosity in the TBCs structure. A very slight rise of Young's modulus has been observed at initial drip coating times: the Young's modulus in the top region rises from 60.56 to 66.30 GPa when drip coating time increases from 0 to 2. However, a sudden increase in Young's modulus occurs when drip coating time increases to 3, which is coincident with the rapid increase of Al_2_O_3_ packing density in the column gap (Figure 3d).

The materials probed by micro indentation span several columns. As a result, the Young's modulus determined by micro indentation includes the contribution from both individual columns and inter‐columnar gap. To understand each contribution separately, nano indentation was used to carry out site‐specific measurements on column center and column gap, respectively. A total of 160 indents were made on the cross section of TBCs with corresponding indentation depths of no >200 nm. After indentation, SEM was used to help pick out indents made on the column center and the column edge. Results of those selected indents are shown in Figure 6b. For unprotected TBCs, column edge has a lower Young's modulus than the column center due to its porous feathery morphology. The Young's modulus of column center is similar for unprotected TBCs and Al_2_O_3_‐protected TBCs. However, a significant increase of Young's modulus has been observed at the column edge for Al_2_O_3_‐protected TBCs. As expected, Al_2_O_3_‐protected TBCs has a much higher Young's modulus at column edge due to Al_2_O_3_ coverage: it is more than twice higher than that of unprotected TBCs (≈34.7 GPa), and it increases with drip coating time. Thus, it can be seen the injection of Al_2_O_3_ brings significant increase to the Young's modulus of column edge but has little impact on column center.

**Figure 7:**
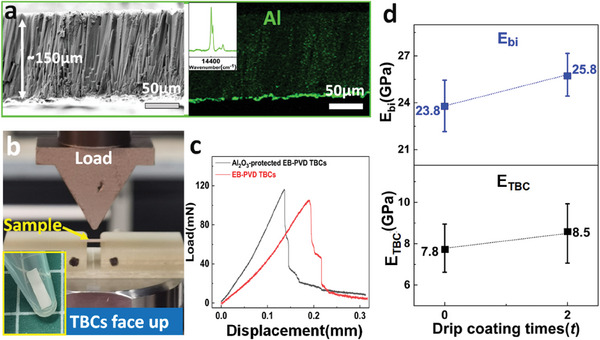
Illustration of the three‐point beam test configuration and Young's modulus of free‐standing TBCs. a) fractured cross‐section and elemental (Al) mapping of ceramic bilayer (TBC+TGO), with an inset of a PLPS spectrum taken from the TGO side of the ceramic bilayer; b) illustration of the three‐point beam test configuration; c) examples of load‐displacement curves for ceramic bilayer (TBC+TGO); d) comparison of Young's modulus (*E*_TBC_) between the unprotected TBCs and Al_2_O_3_‐protected TBCs. The Young's modulus of the TBC layer is deconvoluted from that of the ceramic bilayer (*E*_bi_) using the composite beam model. The Al_2_O_3_‐protected EB‐PVD TBCs is obtained by drip coating two times. The error bars represent the standard deviation.

To compare the global elastic behavior of Al_2_O_3_‐protected TBCs and unprotected TBCs, three‐point beam bending tests have been conducted, and the results are shown in **Figure** **7**. Based on the cross‐sectional element (Al) map (Figure 7a) and the PLPS spectrum obtained from the backside (TGO side) of the ceramic bilayer (inset in Figure 7a), it has been confirmed that the free‐standing TBC is a ceramic bilayer (TBC+TGO) with a thickness of ≈150 µm. Figure 7b presents the three‐point beam bending test configuration. The load‐displacement curves of Al_2_O_3_‐protected TBCs and unprotected TBCs are quite similar, as depicted in Figure 7c, indicating that the built‐in Al_2_O_3_ has minimal effect on the overall mechanical behavior of EB‐PVD TBCs. By analyzing the load‐displacement curves, the Young's modulus of the bilayer beam (*E_bi_*) was determined, and subsequently, the Young's modulus of the TBC (*E_TBC_*) was calculated from *E_bi_* using the composite model mentioned in Section 2.3.

Figure 7d presents the Young's modulus of the bilayer beam (*E_bi_*) and TBC (*E_TBC_*). It is observed that for both Al_2_O_3_‐protected TBCs and unprotected TBCs, the Young's modulus of the bilayer beam (*E_bi_*) is approximately three times higher than the single layer of TBC (*E_TBC_*), indicating the significant role of the TGO layer in the Young's modulus of the bilayer beam. Therefore, the application of the composite model is essential for accurately determining the Young's modulus of the TBC layer in this study. Moreover, it is noted that the *E_TBC_* of Al_2_O_3_‐protected TBCs (≈8.5Gpa) is ≈9% higher than the unprotected TBCs (≈7.8Gpa), suggesting a minor impact of the injected Al_2_O_3_ on the global elastic modulus of TBCs. Furthermore, the Young's modulus of TBC (*E_TBC_*) obtained through the beam bending test is significantly lower than that obtained through micro‐indentation, while the Young's modulus measured by the two techniques shows similar evolution with the injection of Al_2_O_3_. This discrepancy in modulus is mainly attributed to the different measuring length scales. Therefore, by combining the results of micro‐indentation, nano‐indentation, and three‐point beam bending tests, we can conclude that when the drip coating time is 2, the injection of Al_2_O_3_ can have a minor impact on the Young's modulus of TBCs, both at the scale of several columns and throughout the entire coating. Meanwhile, at the individual column level, the injected Al_2_O_3_ significantly increases the Young's modulus at the column edges, while having minimal impact on the column center.

#### 3.2.2 Sintering Effect

The injection of Al_2_O_3_ in EB‐PVD TBCs modifies its inter‐columnar structure, which is expected to affect the sintering behavior of TBCs. In this work, microstructural evolution and Young's modulus in thermal exposure were studied to characterize the effect of Al_2_O_3_ on the sintering of TBCs. Young's modulus determined by micro and nano indentations has been used to reflect the sintering behavior of several columns and individual columns of TBCs, respectively. Young's modulus determined by three‐point bending is used to understand the sintering of TBCs at the macroscopic scale. The indentation measurements were all conducted in the top regions of TBC cross‐sections (≈60 µm from the TBC surface).

**Figure 8:**
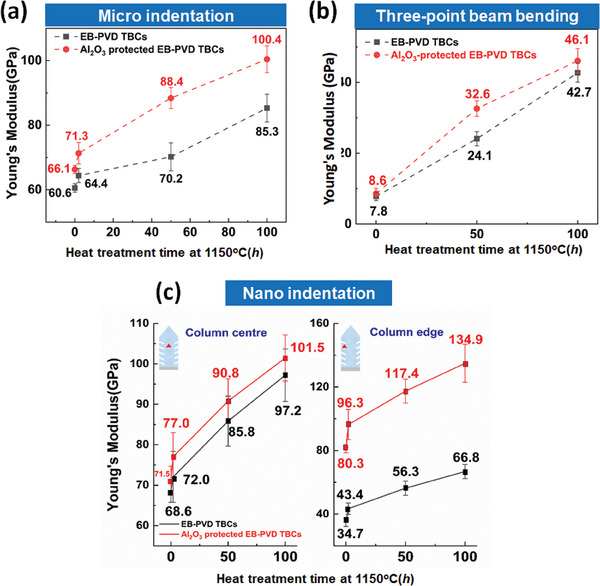
Young's modulus for EB‐PVD TBCs with and without Al_2_O_3_ protection after sintering at 1150 °C as a function of sintering duration (time = 0, 2, 50, and 100 h). a) Young's modulus of EB‐PVD TBCs with and without Al_2_O_3_ protection measured by micro indentation; The micro indentation measurements cover multiple columns and inter‐columnar gaps b) Young's modulus of the free standing EB‐PVD TBCs with and without Al_2_O_3_ protection measured by three‐point beam bending; c) Young's modulus of column center and edge measured by nano indentation. EB‐PVD TBCs with Al_2_O_3_ protection is obtained by drip coating two times, and the measurement areas of indentation are located at the top regions of the coating cross sections. The error bars represent the standard deviation.

**Figure** **8**a,b plot the Young's modulus of the TBCs measured by micro‐indentation and three‐point beam bending as a function of heat treatment time at 1150 °C. While the two sets of data are not comparable in absolute values, the Young's modulus measured by the two techniques shows similar evolution with thermal exposure. The Young's modulus of both types of TBCs (unprotected TBC and Al_2_O_3_‐protected TBC) increases with heat treatment but shows the highest increase rate in the initial 2 h. Within the initial 50 h thermal exposure, the growth rate of Young's modulus of the Al_2_O_3_‐protected TBCs is higher than that of the unprotected TBCs. However, the relationship is reversed after long term heat treatment (*t* > 50 h) in which the growth rate of Young's modulus for Al_2_O_3_‐protected TBCs is 20−28% lower than that of unprotected TBCs. The findings suggest that built‐in Al_2_O_3_ accelerates sintering of TBCs at the initial sintering stage but slows down the process during long term heat treatment (*t* > 50 h).

Figure 8c shows the Young's modulus of column center and edge obtained by nano indentations. It can be found that column center shows similar sintering behavior for both TBCs, while Al_2_O_3_‐protected TBCs has a relatively higher sintering rate at the column edge. The overall growth rate of Young's modulus of column edge is ≈0.55 GPa h^−1^ for Al_2_O_3_‐protected TBCs and is almost twice as higher than that of unprotected TBCs (≈0.32 GPa h^−1^). The evolution of Young's modulus of column center and edge indicates a higher sintering rate of the individual column in Al_2_O_3_‐protected TBCs than that in unprotected TBCs. On the other hand, we recall that the micro‐indentation measurements (Figure 8a), which cover multiple individual columns and inter‐columnar gaps, indicate a lower sintering rate for Al_2_O_3_‐protected TBCs during long‐term thermal treatment (*t* > 50 h). The difference between micro‐indentation and nano‐indentation results suggests that the sintering rate of the inter‐columnar gaps, probed by micro indentation, is lower in Al_2_O_3_‐protected TBCs compared to the unprotected TBCs. This lower sintering rate of inter‐columnar gap counterbalances the higher sintering rates of the individual columns, which thereby results in an overall lower sintering rate in Al_2_O_3_‐protected TBCs during long‐term thermal treatment.

**Figure 9:**
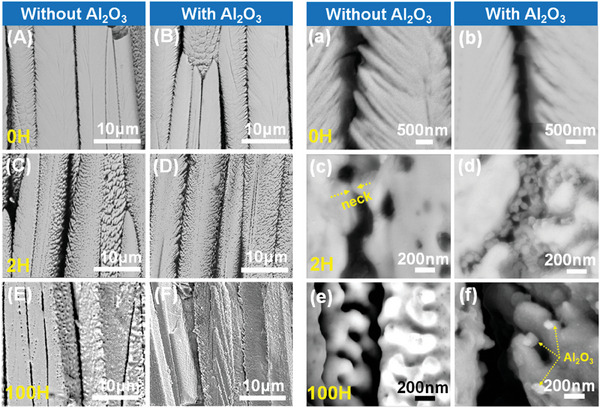
Cross sectional microstructures in different magnifications for TBCs without and with Al_2_O_3_ protection after sintering at 1150 °C for 0 h: (A,a,B,b); 2 h: (C,c,D,d) and 100h: (E,e,F,f).

**Figure 10:**
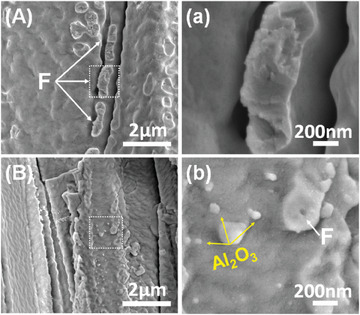
Necks between columns revealed in the fractured cross‐section after exposure at 1150 °C for 100 h. A,a): TBCs without Al_2_O_3_ protection; B,b): TBCs with Al_2_O_3_ protection. Fractured necks, F, are indicated.

**Figure** **9** compares the cross‐sectional microstructure of both TBCs at different sintering durations (*t* = 0, 2 and 100 h). For unprotected TBCs, neck formation starts at the initial sintering stage (*t* = 2 h) (Figure 9c). With the increase of thermal treatment time, the typical feathery feature of EB‐PVD TBCs gradually disappears and is substituted by a smooth surface (Figure 9E,e). Rows of necks (indicated as F) were formed at the inter‐columnar gap after thermal treatment at 1150 °C for 100 h (**Figure** **10**A,a). In contrast, few necks have been observed in Al_2_O_3_‐protected TBCs (Figure 10B,b). Nanoscale Al_2_O_3_ spheres can be observed at both intra‐columnar and inter‐columnar gaps (Figure 9d), while the feathery features are still distinguishable for Al_2_O_3_‐protected TBCs after exposure at 1150 °C for 100 h (Figure 9f).

**Figure 11:**
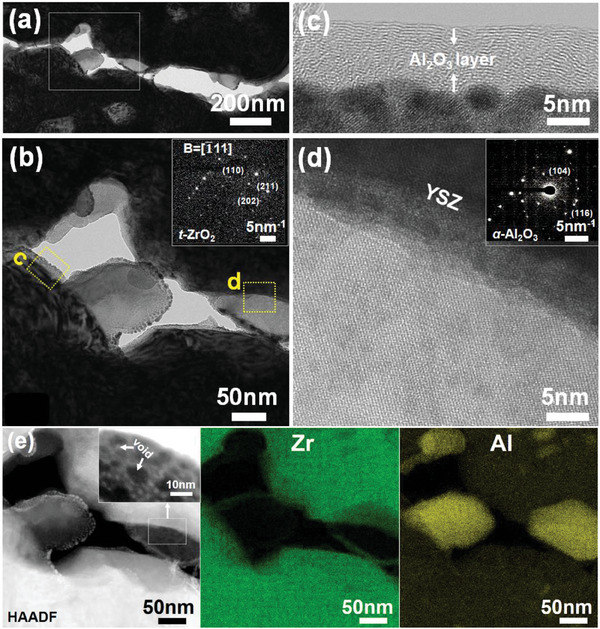
Microstructure and element distribution of TBCs with Al_2_O_3_ protection after exposure at 1150°C for 100 h. a) bright field TEM image of column gap; b) zoomed‐in image of the white box in image (a); c) zoomed‐in image of the yellow box in image (b); d) HRTEM image with corresponding SAED pattern of the interfacial region between Al_2_O_3_ and YSZ column, obtained from the yellow box (d) in image (b); e) HAADF image and EDS map of image (b).

To investigate the role Al_2_O_3_ plays in the inter‐columnar gap, a thin lamella has been prepared from the cross‐section of sintered Al_2_O_3_‐protected TBCs (1150 °C, 100 h) by FIB and then analyzed by STEM, TEM, and EDS. It can be found that the adjacent columns have been separated by spherical Al_2_O_3_ particles, with abundant voids well‐preserved at the inter‐columnar gap (**Figure** **11**a,b). Clear interface is exhibited between the YSZ column and Al_2_O_3_ spheres, with no interaction or diffusion regions observed (Figure 11d). Only *t*‐ZrO_2_ and α‐Al_2_O_3_ phases have been observed at the region near the YSZ/Al_2_O_3_ interface, based on its diffraction pattern shown in the inset image of Figure 11d. The microstructural analysis indicates that the Al_2_O_3_ on the columns wall inhibits column merging and supports the indentation results that the sintering of inter‐columnar gaps is slowed down in Al_2_O_3_‐protected TBCs after long term heat treatment.

### 3.3 CMAS Resistance

#### 3.3.1 CMAS Attack Behavior

**Figure 12:**
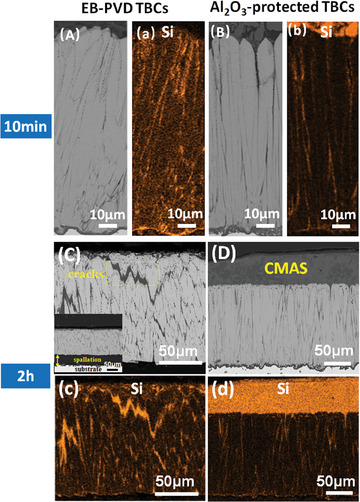
Microstructure and Element (Si) distribution of EB‐PVD TBCs and Al_2_O_3_‐protected TBCs after CMAS attack at 1250 °C for 10 min and 2 h. Cross‐sectional SEM image and Si map of A,a): EB‐PVD TBCs after CMAS attack at 1250 °C for 10 min; B,b): Al_2_O_3_‐protected EB‐PVD TBCs after CMAS attack at 1250 °C for 30 min; C,c): EB‐PVD TBCs after CMAS attack at 1250 °C for 2 h; D,d): Al_2_O_3_‐protected EB‐PVD TBCs after CMAS attack at 1250 °C for 2 h.

TBCs with and without Al_2_O_3_ protection were exposed to CMAS attack at 1250 °C for different durations (*t* = 10 min, 30 min and 2 h) to study their degradation behavior. It is expected that a higher content of Al_2_O_3_ in TBCs structure results in better CMAS resistance but an excessive amount of Al_2_O_3_ would cause a potential loss of strain tolerance to the coating (Figure 6). Therefore, Al_2_O_3_‐protected TBCs (drip coated two times) has been chosen for CMAS attack testing since it has a relatively low Young's modulus (Figure 6) and a small amount of Al_2_O_3_ in TBCs structure. Fast penetration has been observed in both TBCs: TBCs with and without Al_2_O_3_ protection were infiltrated through by CMAS melt for no >10 min, as Si has been identified at TBCs bottom (**Figure** **12**a,b).

**Figure 13:**
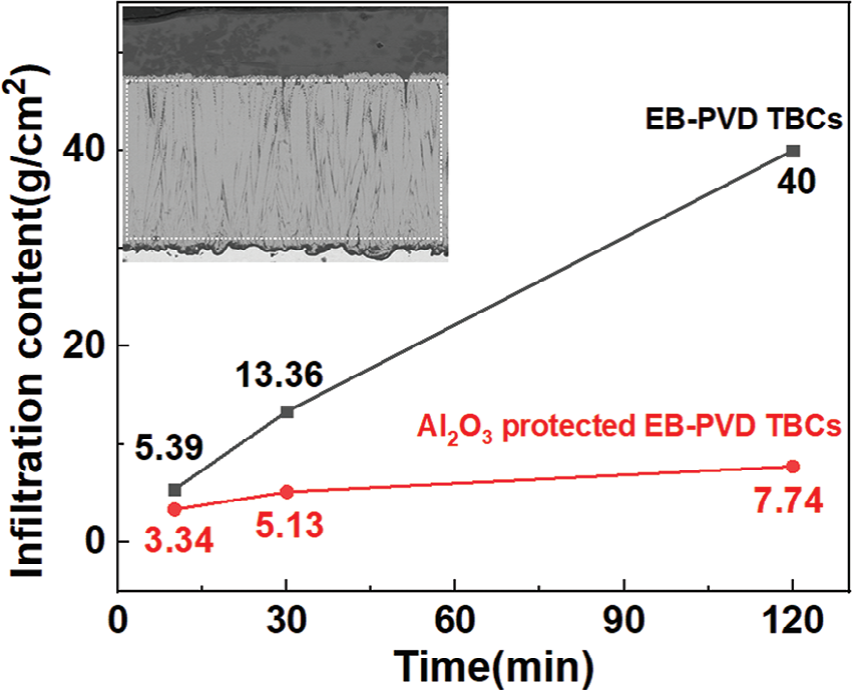
CMAS melt content in EB‐PVD TBCs and Al_2_O_3_‐protected TBCs after exposure to CMAS attack for different lengths of time (time = 10 min, 30 min, and 2 h). EDS scan box was performed on coating cross section to obtain elements (Ca, Mg, Al, Si, Zr, Y, and O) weight ratios and the results were used to calculate the content of CMAS melts in TBCs structure.

However, the contents of the infiltrated CMAS melt show a significant difference between TBCs with and without Al_2_O_3_ protection. Here, the proportion of melt constituents was assumed to be the same as that of the original deposit (35CaO–10MgO–7Al_2_O_3_–48SiO_2_), thereby exhibiting a fixed ratio between the melt and its constituents. By quantifying the concentration of Ca (in a way similar to the aforementioned Al quantification process), we consequently obtain the melt content in TBCs structure. **Figure** **13** compares the content of the infiltrated melt in TBCs with and without Al_2_O_3_ protection at different times (*t* = 10 min, 30 min and 2 h), in which significantly higher CMAS amount and infiltration rates have been observed in the unprotected TBCs. The amount of the infiltrated CMAS is more than five times higher in the unprotected TBCs than that of Al_2_O_3_‐protected TBCs after CMAS attack for 2 h. The CMAS infiltration rate in unprotected TBCs is almost constant, while it decreases with the CMAS attack time for Al_2_O_3_‐protected TBCs. In addition, the CMAS infiltration rate for Al_2_O_3_‐protected TBCs is <1/10 of that of unprotected TBCs in long‐term CMAS attack (*t* >30 min). Those results indicate effective suppression of melt infiltration in Al_2_O_3_‐protected TBCs.

**Figure 14:**
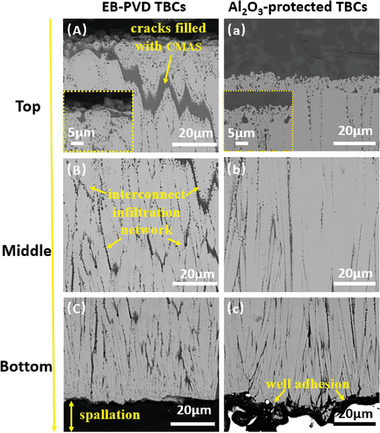
Microstructure of EB‐PVD TBCs and Al_2_O_3_‐protected TBCs after CMAS attack at 1250 °C for 2 h. A): top region of EB‐PVD TBCs, with an inset of magnified image; B): middle region of EB‐PVD TBCs; C): bottom region of EB‐PVD TBCs; a): top region of Al_2_O_3_‐protected EB‐PVD TBCs, with an inset of magnified image; b): middle region of Al_2_O_3_‐protected EB‐PVD TBCs; c): bottom region of Al_2_O_3_‐protected EB‐PVD TBCs.

**Figures** 12 and **14** present the microstructure and EDS map of CMAS attacked samples. After CMAS attack for 2 h, no CMAS was observed on the unprotected TBCs (Figure 12c). The ceramic coating fell off from the substrate, and numerous transverse cracks filled with CMAS melt (Figure 12c) have been found across the EB‐PVD columns. In addition, severe degradation of column tips has been observed. The column tip decomposed to randomly oriented grains and small spherical grains (inset of Figure 14A). Based on XRD and EDS semi‐quantitative analysis, those randomly oriented grains were identified as tetragonal Y‐lean ZrO_2_ with solid solution of Ca and Si, which were formed through the interaction between CMAS/TBCs. Additionally, the small spherical grains were monoclinic ZrO_2_, generated through the phase transformation of tetragonal Y‐lean ZrO_2_ grains during cooling.^[^ ^42^, ^43^ ^]^

**Figure 15:**
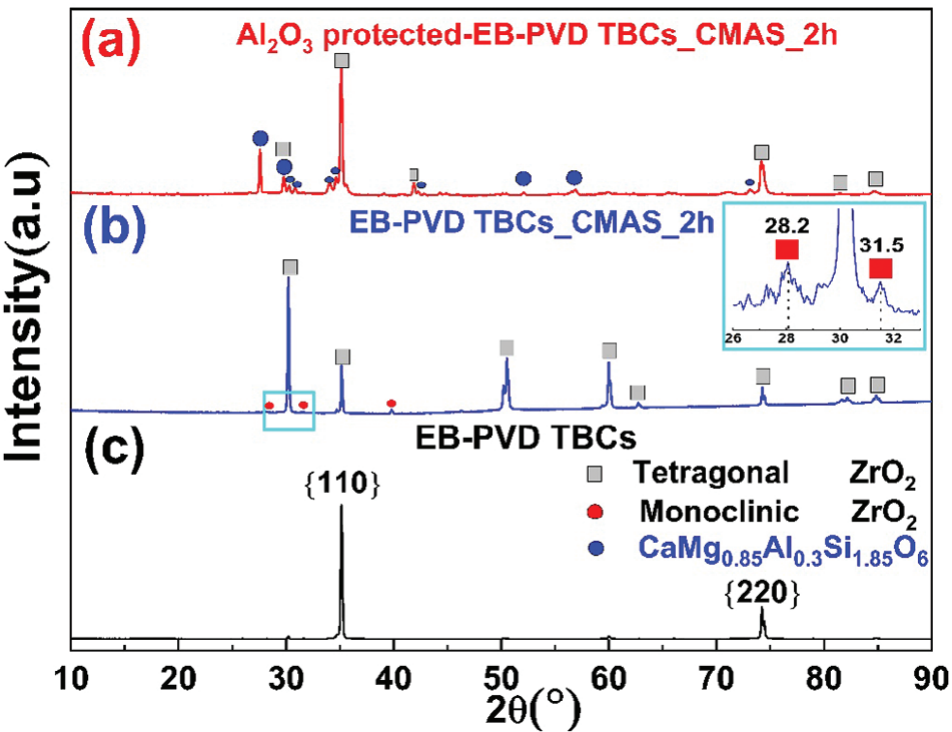
X‐ray diffraction patterns of a) CMAS attacked Al_2_O_3_‐protected EB‐PVD TBCs at 1250 °C for 2 h; b) CMAS attacked EB‐PVD TBCs at 1250 °C for 2 h; c) EB‐PVD TBCs.

However, less damage has been observed for Al_2_O_3_‐protected TBCs after CMAS attack. The majority of CMAS was resisted outside the ceramic coating, forming a thick glass layer composed of numerous CMAS self‐crystallizations (Figure 12D). The phase structure of CMAS self‐crystallization is confirmed as anorthite by XRD (**Figure** **15**), and its composition is close to CaM_g1.2_Al_0.16_Si_2.28_O_6.98_ based on EDS semi‐quantitative analysis. Besides, the ceramic coating is well bonded to the substrate, and no vertical cracks have been observed in the coating structure (Figure 12d). Moreover, much fewer monoclinic Y‐depleted zirconia grains have been generated in TBCs structure. The structural degradation of YSZ columns mainly happens within the upper 20 µm region (Figure 13a). The middle and bottom areas of the column have retained their initial structure to a large extent (Figure 13b,c). In addition, these morphology results are well supported by the XRD data. In Figure 15 we can find that the diffraction peaks for the unprotected TBCs after CMAS attack (Figure 15b) are different from that before CMAS attack (Figure 15c). Peaks ascribed to monoclinic ZrO_2_ phase have been observed in TBCs without Al_2_O_3_ after CMAS attack. These findings indicate significant destruction of the original EB‐PVD texture at the top surface of TBCs, which is replaced by randomly oriented small crystals. In addition, no signs of CMAS‐related phases were detected as a result of the complete infiltration of CMAS melt in the unprotected TBCs. However, for the Al_2_O_3_‐protected TBCs, the zirconia peaks (Figure 15a) still look very similar to the original TBCs, which means much less damage has been brought in after CMAS attack. Meanwhile, CMAS‐related phase (CaMg_0.85_Al_0.3_Si_1.85_O_6_) has been observed as a result of the crystallization of surficial CMAS remains, indicating the effective protection from CMAS infiltration in TBCs with Al_2_O_3._

#### 3.3.2 Reaction Product

**Figure 16:**
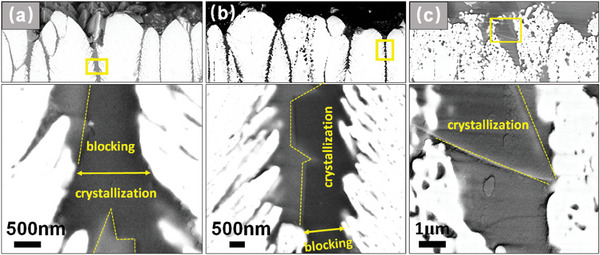
Microstructure of the reaction products in the tip regions of Al_2_O_3_‐protected EB‐PVD TBCs after CMAS attack at 1250 °C for a) 10 min; b) 30 min; c) 2 h. The magnified images of column gaps are taken from the marked regions by yellow rectangles.

To understand why Al_2_O_3_‐protected TBCs is able to withstand CMAS attack, both CMAS remain/TBCs and column wall/CMAS glass interfaces were studied in detail. The morphology of CMAS remains/TBCs interface region was investigated by SEM. After CMAS attack for 10 min, strip‐shaped reaction products were produced in a region close to the surface of TBCs (**Figure** **16**a). These products were identified as anorthite by XRD (Figure 15), effectively blocking the infiltration pathway of CMAS melt. With extension of CMAS attack time, the reaction products are still well preserved at the entrance of the column gap, while the feathery structure of EB‐PVD TBCs gradually diminished (Figure 16b,c).

**Figure 17:**
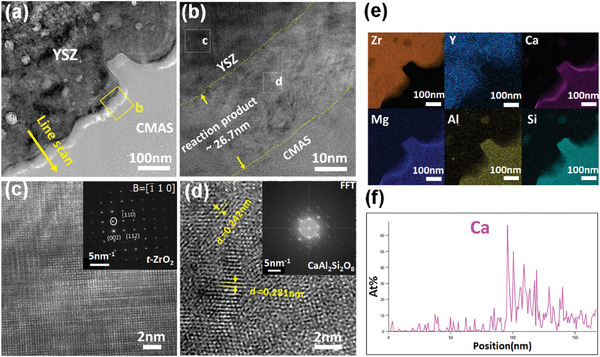
Microstructure and elements distribution of Al_2_O_3_‐protected EB‐PVD TBCs after CMAS attack at 1250 °C for 2 h. a) Bright‐field TEM image of YSZ/CMAS interfacial region; b) Zoom‐in image of the yellow box in image (a); c) HRTEM image with corresponding SAED pattern of YSZ column, obtained from the white box (c) in the image (b); d) HRTEM image with corresponding SAED pattern of reaction layer, obtained from the white box (d) in the image (b); e) EDS map of image (a) and (f) EDS line scan of the interfacial region, marked by the yellow arrow in image (a).

A thin lamella across the column wall/CMAS glass interface was prepared by FIB and then analyzed by STEM, TEM, and EDS. A continuous dense reaction layer with a thickness of tens of nanometers has been formed on the YSZ column after CMAS attack (**Figure** **17**a). This reaction layer is identified as anorthite polycrystalline with a composition close to Ca_2.3_Mg_1.1_AlSi_2.4_O_10.2_, according to EDS and HRTEM analysis (Figure 17d). Tightly adherence has been observed between the reaction layer and YSZ column, as shown in Figure 17b.

To evaluate the protective effect of this reaction layer, both elemental and phase analysis have been conducted on the YSZ column after CMAS attack at 1250 °C for 2 h. EDS map and line scan have been performed near the interfacial region of CMAS/YSZ column (Figure 17e,f). An EDS line scan was conducted along the yellow arrow shown in Figure 17a. Enrichment of Ca has been observed at the CMAS/YSZ interface due to the existence of a reaction layer on the YSZ column, while few CMAS was detected inside the YSZ column (Figure 17e,f). Figure 17c presents the diffraction pattern and HRTEM image of the corroded YSZ column. The phase structure of corroded YSZ column is identified as tetragonal ZrO_2_, indicating few *t*‐*m* phase transformation has been induced in YSZ column by CMAS attack. Those results suggest the reaction layer on YSZ column mitigates CMAS infiltration toward TBCs column and preserves the integrity of column structure largely.

## 4 Discussion

### 4.1 CMAS Resistance Improvement

We have confirmed the Al_2_O_3_‐protected TBCs exhibit superior resistance against CMAS attack. Apparent suppression of CMAS infiltration and less structural damage have been achieved in Al_2_O_3_‐protected TBCs. The CMAS‐resistant mechanism for Al_2_O_3_‐protected TBCs is discussed below.

The molten CMAS is prone to penetrate through the inter‐columnar gaps of EB‐PVD TBCs via capillary force.^[^ ^44^, ^45^ ^]^ For Al_2_O_3_‐protected TBCs, the vulnerable infiltration pathways were decorated with nanoporous Al_2_O_3_ (Figure 5b), which physically narrows the pathways for CMAS to penetrate. Most importantly, the nanoporous Al_2_O_3_ can serve as both a melt trap and an Al reservoir during CMAS attack. When molten CMAS flows through, the nanoporous Al_2_O_3_ can spontaneously suck it in via capillary force and restrict its fluidity. The enlarged contact area between the trapped melt and the nanoporous Al_2_O_3_ initiates fast dissolution of Al_2_O_3_ in the melt, which thereby shifts the composition of CMAS melt to a field easy to crystallize^[^ ^23^ ^]^ and promotes the fast crystallization of Al‐enriched CMAS at the infiltration pathway. As a result, strip‐shaped reaction products were produced at the entrance of the column gap after CMAS attack for 10 min (Figure 16a), while a continuous reaction layer was formed on YSZ column (Figure 17a).

The strip‐shaped reaction products play a role as a pathway blocker at the entrance of column gap, which resists CMAS penetration at the front of the infiltration pathway. However, those blockers (reaction products) alone are not enough to achieve a long‐standing blocking effect. Side path along the column wall and transversal path toward EB‐PVD column could be created due to YSZ dissolution and grain boundary penetration of CMAS melt. Those new infiltration pathways could undermine the blocking effect of the reaction products at the entrance of column gap and re‐activate melt infiltration in TBCs structure. It is evident in Figure 12C that broadened column gap and transversal channels have been generated in the unprotected TBCs after CMAS attack. Those channels create an interconnected infiltration network in TBCs structure (Figure 13B), largely promoting melt injection. However, the creation of the interconnect infiltration network has been prevented in Al_2_O_3_‐protected TBCs. This is because a continuous crystallization layer was formed on the column wall due to the fast crystallization of nanoporous Al_2_O_3_‐trapped CMAS on column wall (Figure 17b). This crystallization layer protects the column structure against both YSZ dissolution and infiltration pathway development. Consequently, few new paths were carved out in Al_2_O_3_‐protected TBCs and no further CMAS infiltration can be easily achieved. Moreover, the decoration of a crystallization layer on the column wall also modifies the wettability of the infiltration pathway. The presence of anorthite crystallization layer, which possesses a lower surface energy compared to YSZ,^[^ ^46^ ^]^ is expected to result in a larger intrinsic contact angle between the molten CMAS and the crystallization layer. This indicates a reduced wettability of the molten CMAS on the crystallization layer compared to the bare YSZ column, which consequently results in constrained fluidity of molten CMAS within Al_2_O_3_‐protected TBCs. As shown in Figure 14, we can see much less CMAS melt has been infiltrated in Al_2_O_3_‐protected TBCs in comparison to the unprotected TBCs and the infiltration rate of CMAS melt in Al_2_O_3_‐protected TBCs decreases with the increase of CMAS attack time, due to the combined effect of crystallizations at both column gap entrance and on column wall.

In addition to suppression of melt infiltration, those crystallizations inhibit thermochemical degradation of EB‐PVD TBCs. It is well known that Y_2_O_3_ depleted ZrO_2_ grains are prone to be produced during the dissolution‐precipitation process, due to different solubility of Zr^4+^ and Y^3+^ in CMAS melt.^[^ ^47^ ^]^ Upon cooling, those Y‐lean ZrO_2_ grains tend to go through phase transformation (tetragonal to monoclinic phase), which is associated with ≈4.5% volume expansion and irreversible damage to TBCs structure. For Al_2_O_3_‐protected TBCs, the crystallization layer on individual column isolates it from CMAS melt, which in turn avoids the dissolution of YSZ column and maintains the integrity of column structure. In contrast to the unprotected TBCs, much less monoclinic phase and structural degradation have been found in Al_2_O_3_‐protected TBCs after CMAS attack (Figures 13a–c and 15). Therefore, we can see much less CMAS melt has been infiltrated in Al_2_O_3_‐protected TBCs compared to the unprotected TBCs, and the infiltration rate of CMAS melt in Al_2_O_3_‐protected TBCs decreases with the increase of CMAS attack time, due to the combined effect of crystallizations at both column gap entrance and on the column wall.

### 4.2 Young's Modulus

The introduction of a built‐in α‐Al_2_O_3_ protection network is inevitable to raise the elastic modulus of TBCs structure (Figure 6) attributed to coating porosity reduction and introduction of stiffer Al_2_O_3_ (*E*_Al2O3_ ≈386 GPa;^[^ ^48^ ^]^ *E*_YSZ_ ≈205 GPa). This work investigated the effect of injected Al_2_O_3_ on the Young's modulus of TBCs, and the effect of drip coating times on the Young's modulus of TBCs.

In general, the injection content of Al_2_O_3_ determines the Young's modulus of Al_2_O_3_‐protected TBCs in macroscopic scale, while the distribution of Al_2_O_3_ within the TBCs structure plays a specific role in the Young's modulus of each component (e.g., YSZ column and column gap). Based on the morphology results, we've found that the majority of Al_2_O_3_ is attached to the column wall in a nanoporous morphology rather than accumulating at the column gap after drip coating (Figure 5). This distribution of Al_2_O_3_ in TBCs maintains the strain compliance of the EB‐PVD TBCs structure, and identifies the YSZ column as the component experiencing the most substantial modifications upon the injection of Al_2_O_3_. For the individual YSZ column, the injection of Al_2_O_3_ mainly affects the Young's modulus of the column edge, while its impact on the column center is negligible according to the nano indentation results (Figure 6b). Consequently, it can be concluded that the injection of Al_2_O_3_ primarily decorates the column wall of TBCs, resulting in a significant impact on the Young's modulus specifically at the column edge in EB‐PVD TBCs.

Based on the results of micro indentation, it has been found that the Young's modulus of TBCs increases with drip coating time (Figure 6a), but the influence of each drip coating on the Young's modulus of TBCs is not equal. The initial drip coating stages (*t* = 1 and 2) have a minor impact on the Young's modulus of Al_2_O_3_‐protected TBCs, while a sudden increase in Young's modulus occurs when drip coating time increases to 3. The growth rate of Young's modulus slows down after drip coating for three times. The underlying mechanism behind the varying impact of each drip coating on the Young's modulus is elucidated below. The evolution of Young's modulus is determined by the injection content of Al_2_O_3_, which largely depends on the injection volume of AlCl_3_ sol (Figure 3c). With other parameters being fixed (e.g., temperature and drip coating process), the injection volume of AlCl_3_ sol is determined by the size and surface topography of the infiltration channel. The introduction of Al_2_O_3_ not only narrows down the channel but also modifies the channel surface with nanoporous structure, which consequently results in a pair of opposite effects on AlCl_3_ sol injection. For a given inter‐columnar gap in the as‐deposited TBC, it is surface topography of infiltration channel that dominates AlCl_3_ sol injection at the initial drip coating stage: the nano porous Al_2_O_3_ on the channel surface accelerates AlCl_3_ sol injection by capillary imbibition.^[^ ^49^ ^]^ However, with further increase in drip coating time, the infiltration channel is gradually clogged by Al_2_O_3_, especially the entrance of infiltration channel (column groove) as shown in Figure 4a. As a result, the size effect of infiltration channel plays a major role in later drip coating, which inhibits AlCl_3_ sol injection. Due to the joint effects of channel size and surface topography, an unequal impact has been placed on coating's Young's modulus by each drip coating. This finding provides guidance on how to build up TBCs structure with controllable Young's modulus in the future.

Furthermore, a higher injection content of Al_2_O_3_ improves the resistance to CMAS attack but tends to decrease the strain tolerance of TBCs and potentially its lifetime due to the increased Young's modulus. Therefore, it is important to strike a balance between the benefits of improved CMAS resistance and the limitations on strain tolerance. In this work, a minor effect on the Young's modulus of Al_2_O_3_‐protected TBCs was observed after drip coating 2 times, while a significant enhancement in CMAS resistance compared to unprotected TBCs was achieved (Figures 12, 13, 14). This evidence is supported by the global in‐plane modulus of the TBCs layer which increased from 7.8 to 8.6 GPa after drip coating 2 times, as measured through three‐point beam bending (Figure 7d). Additionally, the load‐displacement curves obtained from the beam bending tests (Figure 7c) also indicate minor alterations in the overall mechanical behavior of TBCs after drip coating 2 times. Those findings present an opportunity to break the trade‐off between strain tolerance and CMAS resistance through this novel TBC architecture.

### 4.3 Sintering Effect

**Figure 18:**
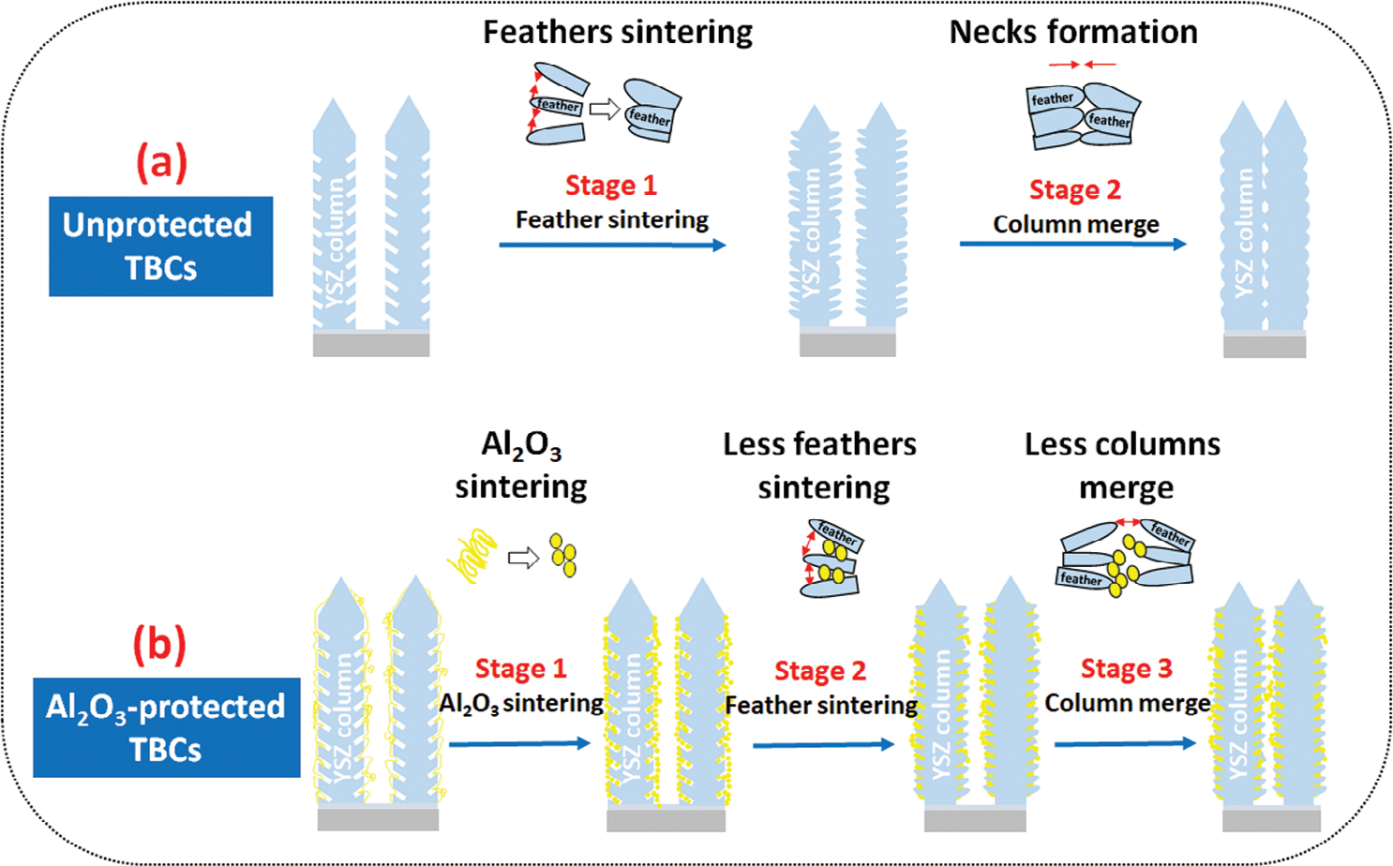
Schematic of the sintering progress for a) unprotected TBCs and b) Al_2_O_3_‐protected TBCs.

The experimental results reveal that the introduction of Al_2_O_3_ accelerates the sintering of TBCs at the initial stage but slows it down during long‐term thermal treatment. A lower growth rate of Young's modulus has been observed in Al_2_O_3_‐protected TBCs in comparison to the unprotected TBCs after exposure at 1150 °C for 50 h (Figure 8). In addition, much fewer necks were formed between the adjacent columns in Al_2_O_3_‐protected TBCs after long‐term sintering (Figure 9f). The primary mechanism of the sintering behavior of Al_2_O_3_‐protected TBCs is explained below, assisted by a schematic shown in **Figure** **18**.

As depicted in Figure 18a, the densification process of EB‐PVD TBCs usually occurs in two stages: 1) feathery sintering, and 2) column merge. First, the typical feathery morphology of individual columns smoothens with the simultaneous development of surface undulations. Then, some of these undulations on adjacent columns grow in amplitude until impinging to span the gap between the columns, which thereby forms rows of local necks along the columns.^[^ ^50^ ^]^ Those necks promote inter‐columnar mass transportation and motivate the sintering together of adjacent columns (Figure 9). In brief, the column merge process is determined by the formation and growth of necks between adjacent columns.

In contrast to the unprotected TBCs, the densification process of Al_2_O_3_‐protected EB‐PVD TBCs occurs in three stages as depicted in Figure 18b: 1) Al_2_O_3_ sintering, 2) feathery sintering, and 3) column merge. Due to the smaller size of nanoporous Al_2_O_3_ than columns feather, sintering of Al_2_O_3_‐protected TBCs takes place on nanoporous Al_2_O_3_ first. At initial sintering stage, the nanovoids (Figure 5b) within the Al_2_O_3_ gradually diminish, initiating a shape change toward a more spherical morphology (Figure 11e), as this configuration helps to minimize the overall surface energy.^[^ ^51^ ^]^ The as‐formed Al_2_O_3_ nano spheres then prevent feather merge due to the immiscibility between Al_2_O_3_ and YSZ,^[^ ^21^ ^]^ which thereby mitigates the development of surface undulations (Figure 9d–f). Owing to the mitigation of surface undulations development on individual column, the probability of neck formation between adjacent columns has been reduced. As shown in Figure 10, much fewer necks were generated between the adjacent columns for Al_2_O_3_‐protected TBCs in comparison to the unprotected TBCs after exposure at 1150 °C for 100 h.

The presence of Al_2_O_3_ spheres not only suppresses neck formation but also modifies the growth behavior of the necks. Due to the immiscibility between Al_2_O_3_ and YSZ,^[^ ^21^ ^]^ the Al_2_O_3_ coated on the column wall prevents the development of necks along the column direction. As shown in Figure 10a,b, the size of neck in Al_2_O_3_‐protected TBCs is much smaller compared to the unprotected TBCs. Furthermore, as the adjacent columns approach each other, each Al_2_O_3_ sphere within the inter‐columnar gap acts as an obstacle, effectively impeding their further movement. This suppression of neck formation and growth mitigates inter‐columnar mass transportation, thereby inhibiting column merging. Consequently, the densification process of Al_2_O_3_‐protected TBCs is impeded during the later stages of sintering, as supported by the three‐point beam bending and micro‐indentation results in Figure 8. Hence, it can be concluded that the involvement of Al_2_O_3_ in TBCs accelerates the initial sintering due to the inherent densification capability of Al_2_O_3_ itself, while slowing down further sintering by preventing column merging (Figure 8a,b).

## 5 Conclusion

In conclusion, we have designed a novel Al_2_O_3_‐protected EB‐PVD TBCs architecture via vacuum‐assisted drip coating (VADC). This architecture involves decorating the YSZ column with nanoporous Al_2_O_3_, resulting in enhanced CMAS resistance. The fast interaction between the nanoporous Al_2_O_3_ and CMAS melt leads to the generation of crystallizations at the infiltration front of CMAS melt (the entrance of column gap and column wall), effectively suppressing melt infiltration and thermochemical degradation of the TBCs structure. Furthermore, this TBCs architecture exhibits sintering resistance as the nanoporous Al_2_O_3_ prevents neck formation and column merge, thereby slowing down the sintering process during long‐term thermal treatment. Importantly, the scalability and relatively low‐cost of the drip coating method make this novel TBCs architecture highly promising for large‐scale industrial production.

## Conflict of Interest

The authors declare no conflict of interest.

## Data Availability

The data that support the findings of this study are available from the corresponding author upon reasonable request.
